# Effects of CB1 receptor negative allosteric modulator Org27569 on oxycodone withdrawal symptoms in mice

**DOI:** 10.1007/s00213-024-06591-z

**Published:** 2024-04-27

**Authors:** Rhianne L. Scicluna, Nicholas A. Everett, Connie J. Badolato, Bianca B. Wilson, Michael T. Bowen

**Affiliations:** 1 Brain and Mind Centre, The University of Sydney, 94 Mallet Street, Camperdown, Sydney, NSW 2050 Australia; 2 Faculty of Science, School of Psychology, The University of Sydney, Sydney, NSW 2006 Australia

## Abstract

**Rationale/Objectives:**

Targeting cannabinoid receptor type 1 (CB1R) has shown promise for treating opioid withdrawal symptoms. This study aimed to investigate the efficacy of a specific CB1R negative allosteric modulator (NAM), Org27569, in reducing both naloxone-precipitated and protracted withdrawal symptoms in oxycodone-dependent mice.

**Methods:**

Mice received escalating doses of oxycodone (9–33 mg/kg IP) or saline twice daily for 9 days, followed by a final dose of oxycodone (33 mg/kg) or saline in the morning of day 9. In one cohort, the impact of Org27569 (3, 10, and 30 mg/kg) on naloxone (10 mg/kg IP) precipitated withdrawal symptoms was assessed. In another cohort, Org27569 (3 mg/kg) effects on the acquisition of conditioned place aversion to naloxone (0.6 mg/kg) precipitated opioid withdrawal, on behaviour following a 7–9-day abstinence period, and on naloxone (0.6 mg/kg) precipitated withdrawal-induced escape behaviour in a novel assay were assessed.

**Results:**

Although Org27569 decreased opioid withdrawal-induced jumping at doses of 10 and 30 mg/kg, these effects were confounded by reduced locomotion. At all doses tested, Org27569 had a modest inhibitory effect on gastrointestinal motility. At the lower dose of 3 mg/kg, which was not confounded by locomotor effects, Org27569 did not impact naloxone-precipitated withdrawal-induced jumping, acquisition of oxycodone withdrawal-induced conditioned place aversion, or naloxone-precipitated withdrawal-induced escape behaviour in a novel assay. A clear protracted opioid withdrawal phenotype was not observed in assays of anxiety-like or social behaviour.

**Conclusions:**

Org27569 effects on negative affective-like symptoms were confounded by locomotor effects and effects on gastrointestinal motility were not opioid withdrawal specific. Further studies are needed in a model that produces a more pronounced protracted withdrawal syndrome.

**Supplementary Information:**

The online version contains supplementary material available at 10.1007/s00213-024-06591-z.

## Introduction

Opioid overdose deaths reached a new peak of over 80,000 people in the USA in 2021 (NIDA 2023). A key contributor to the acquisition and maintenance of opioid use disorder is the withdrawal syndrome, which drives drug seeking through negative reinforcement (Koob 2019). Withdrawal is characterised by an acute phase, representing the first hurdle to recovery, and a protracted phase, which is a significant barrier to longer term abstinence (Koob and Le Moal 2008). Acute opioid withdrawal is characterised by a range of symptoms including somatic, gastrointestinal and negative affective (Wesson and Ling 2003). Protracted withdrawal is characterised by enduring symptoms including a decreased drive to pursue natural rewards, heightened reactivity to stress, difficulty in recognizing and expressing emotions and physical or emotional pain, feelings of malaise, dysphoria, and sleep disturbances, persistent irritability as well as disruptions in brain activity (for review see Welsch et al. 2020).

Although current maintenance therapies like methadone and buprenorphine significantly reduce all-cause mortality (Fullerton et al. 2014; Schuckit 2016), they face issues like high discontinuation rates, relapse, overdose, restricted access, and induction of withdrawal during switching (Kuehn 2012; Bentzley et al. 2015, Fishman et al. 2019; Varshneya et al. 2022). Lofexidine, the only non-opioid therapy for acute opioid withdrawal, is poorly tolerated, with side effects including sleep difficulties, hypotension, dizziness, dry mouth and bradycardia (Gish et al. 2010; Gorodetzky et al. 2017; Fishman et al. 2019). Additionally, no therapeutics specifically target protracted opioid withdrawal, which is associated with increased relapse vulnerability (Koob and Le Moal 2008). There is thus an urgent need for new non-opioid treatments for acute and protracted opioid withdrawal.

Emerging evidence suggests modulation of cannabinoid receptor type 1 (CB1R), might be a viable strategy for treating opioid withdrawal (Scavone et al. 2013). CB1R knock-out mice had less pronounced naloxone precipitated morphine withdrawal symptoms, including somatic, negative affective, and gastrointestinal symptoms (Ledent et al. 1999; Lichtman et al. 2001; Maccarrone et al. 2002). Sub-chronic co-administration of rimonabant, a CB1R inverse agonist, with morphine reduced various withdrawal symptoms such as digging, teeth chattering, penile licking, wet dog shakes, body weight loss, and jumping (Rubino et al. 2000; Mas-Nieto et al. 2001; Trang et al. 2006). However, the effects of acute rimonabant on opioid withdrawal are less consistent, showing no effect (Mas-Nieto et al. 2001), a reduction in some symptoms (Trang et al. 2006), or exacerbation of symptoms (Navarro et al. 1998), depending on the study. In another study, acute administration of AM251, a CB1R antagonist, had no effect on naloxone precipitated withdrawal from morphine in male Sprague Dawley rats, but chronic administration suppressed withdrawal-induced jumping and teeth chattering (Trang et al. 2006).

Conditioned place aversion (CPA) studies showed CB1R inhibition can alleviate negative affective aspects of opioid withdrawal. Acute administration of CB1R antagonists (AM251, AM6527, AM4113) blocked CPA acquisition in rats undergoing morphine withdrawal (Wills et al. 2014). Direct AM251 administration to the central amygdala (Wills and Parker 2016) or bed nucleus of stria terminalis (Wills et al. 2017) also blocked acquisition of withdrawal-induced CPA. However, studies with rimonabant and AM251 must be interpreted with caution as in addition to their effects on CB1R, they are mu opioid receptor antagonists (Seely et al. 2012), which may explain the complex and sometimes conflicting effects reported across these studies. There is thus merit in further exploring whether inhibition of the CB1R may alleviate some withdrawal symptoms in rodents, especially acute inhibition. Further, since the aforementioned studies were conducted only in male rodents, it is unknown what effect CB1R inhibition has in female mice undergoing opioid withdrawal.

Despite the potential efficacy of CB1R antagonists for opioid withdrawal, they are associated with serious side effects, with rimonabant causing psychosis and depression (Moreira et al. 2009). A safer and more clinically viable approach may be negative allosteric modulation (NAM) of the CB1R. Allosteric modulators typically have reduced on-target safety and efficacy issues, greater receptor specificity, and reduced off-target effects, resulting in a better safety profile than antagonists/agonists (Gao and Jacobson 2013). CB1R NAMs have been designated a spot on NIDAs ten most wanted pharmacological mechanisms for the rapid development of therapeutics in response to the opioid crisis (Rasmussen et al. 2019). It has been shown that CB1R NAM, using the GAT358 compound, blocks opioid-induced reward, without producing preference, aversion, unwanted cannabimimetic side effects or attenuating cannabinoid antinociception (Iyer et al. 2022). However, no study to date has examined the effect of selective CB1R NAM for the treatment of acute and protracted opioid withdrawal symptoms.

Cannabidiol (CBD), which has a diverse range of pharmacological actions (Martin et al. 2021), including CB1R negative allosteric modulation (Laprairie et al. 2015), has been reported to alleviate opioid withdrawal symptoms, (Bhargava 1976; Kudrich et al. 2022; Scicluna et al. 2022). We recently reported CBD reduced both naloxone precipitated and spontaneous opioid withdrawal-induced gastrointestinal upset in mice, but did not reduce negative affective symptoms (Scicluna et al. 2022). In another study, CBD inhibited naloxone precipitated morphine withdrawal symptoms in mice, including increased gastric motility and jumping behaviour (Bhargava 1976). A recent study reported acute administration of CBD reduced spontaneous heroin withdrawal-induced anxiety-like behaviour, rearing, rubbing, grooming, jumping, digging and locomotion (Navarrete et al. 2022). However, the mechanism driving CBD effects in these studies is unclear due to its complex pharmacology (Martin et al. 2021).

This study thus examines the efficacy of a specific CB1R NAM, Org27569 (Yang et al. 2022) in treating opioid withdrawal in mice. Org27569 binds to the allosteric cholesterol-binding site of the CB1 receptor (Shao et al. 2019). The compound has been shown to increase the binding of the orthosteric agonist, CP55940, while inhibiting the ability of CP55940 to produce cAMP and internalise CB1R (Price et al. 2005; Baillie et al. 2013; Cawston et al. 2013). Previous studies using Org27569 have shown some effects on addiction relevant behaviours, including reducing consumption of highly palatable foods in rats (Ding et al. 2014), and attenuating cue and drug-prime-induced reinstatement of cocaine and methamphetamine seeking behaviours in rats (Jing et al. 2014). The present study investigates Org27569’s efficacy in reducing naloxone-precipitated and protracted withdrawal symptoms in oxycodone-dependent mice. During the naloxone-precipitated withdrawal phase, we assessed withdrawal-induced gastrointestinal and negative-affective symptoms. In addition to assessing Org27569 effects on withdrawal-induced jumping, an escape behaviour indicative of negative affect during opioid withdrawal (Azizi et al. 2012), we also examined effects on negative affective-like behaviour measured using a novel escape box assay adapted from Gamage (2013). During the protracted opioid withdrawal phase of the study, we investigated the effects of Org27569 on anxiety-like and social behaviour.

## Methods

**Fig. 1:**
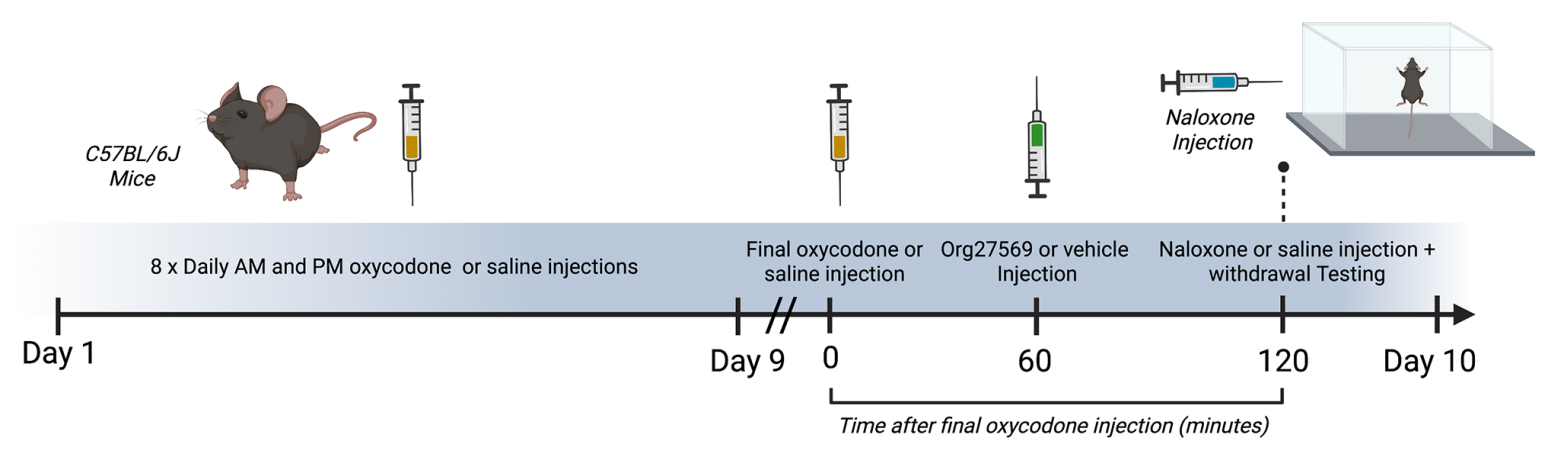
Experimental timeline for acute Org27569 for the treatment of naloxone precipitated oxycodone withdrawal. Mice received escalating doses of oxycodone (9–33 mg/kg IP) or saline, twice daily, for 8 days. On day 9 mice received a final dose of oxycodone (33 mg/kg) or saline. One hour later mice were administered *Org27569* (3, 10 or 30 mg/kg) or vehicle. One hour after treatment mice were injected with 10 mg/kg of naloxone or saline IP and placed the testing arena for a 30 min withdrawal test

**Fig. 2:**
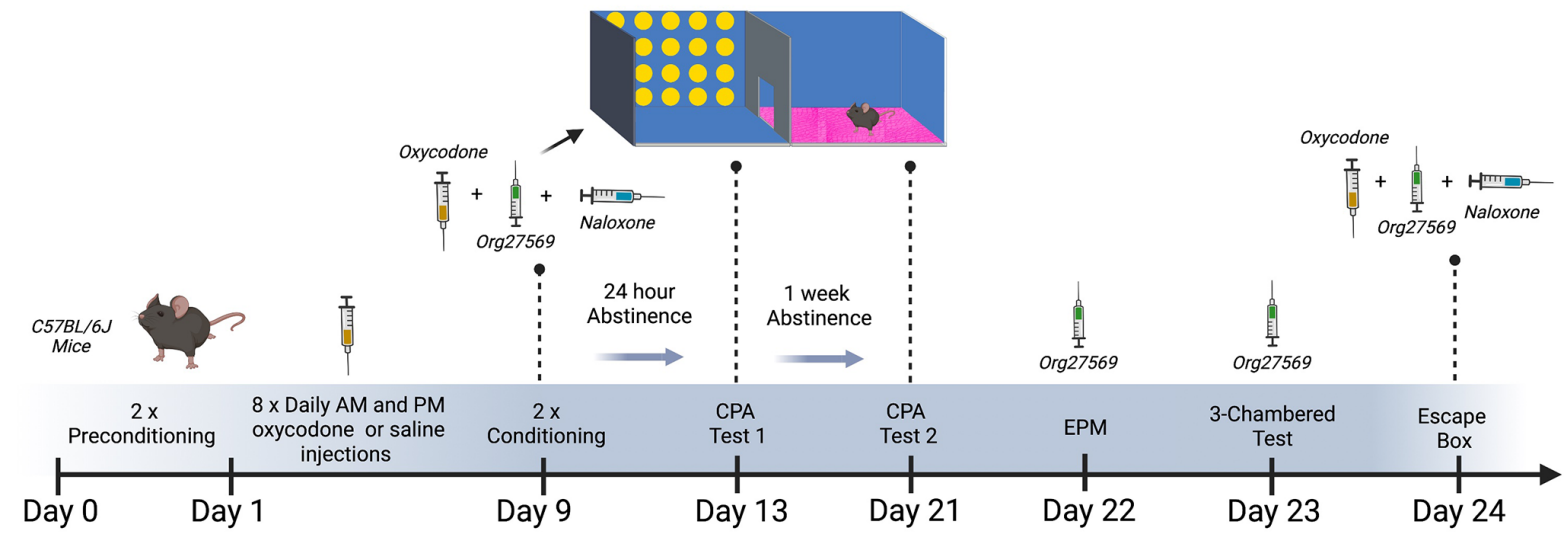
Experimental timeline for acute Org27569 (3 mg/kg) for the treatment of CPA to opioid withdrawal, anxiety like behaviour, social approach, social novelty and escape behaviour. Mice received escalating doses of oxycodone (9–33 mg/kg IP) or saline, twice daily, for 9 days. On day 9 mice received a final dose of oxycodone (33 mg/kg) or saline in the morning. Mice then underwent two rounds of conditioning consisting of a 33 mg/kg oxycodone injection followed by administration of 0.6 mg/kg naloxone or saline injected on alternating days from day 10 to day 12. Mice were then tested for time spent in the withdrawal paired versus saine paired chamber, 24 h into abstinence and 1 week into abstinence on days 13 and 21 respectively. On day 22, mice were treated with 3 mg/kg of Org27569 10 min prior to testing anxiety like behaviour in the EPM. On day 23, mice were treated with 3 mg/kg of Org27569 10 min prior to testing sociability and 15 min prior to testing social novelty in the 3 chambered social approach test. Mice received an injection of 33.3 mg/kg of oxycodone on the evening of day 23. On day 24, mice received an injection of 33.3 mg/kg oxycodone IP followed by 0.6 mg/kg Naloxone IP 2 h later and were then immediately placed in the escape box. Mice received either 0 or 3 mg/kg of Org27569 IP, 10 min prior to naloxone

Timeline of procedures is depicted in Figs. 1 and 2.

### Subjects

Subjects were 97 male and 38 female C57BL/6J mice (9–10 weeks, 17–26 g at commencement of oxycodone administration). Since there were no marked sex differences in the initial precipitated withdrawal experiment, only males were used for subsequent studies to reduce the number of animals used. Individual data points on each figure denote number of mice in each condition. Mice were housed in groups of 4–5 in ventilated cages in a 22^o^C ± 1 and 55%±1 humidity room on a 12-hour light-dark cycle (lights on 6am), with access to food and water *ad libitum*. All testing took place during the light cycle. Mice were given at least 10 days to acclimatise to the facility before the commencement of studies, including two handling sessions. All experimental procedures were approved by University of Sydney Animal Ethics Committee.

### Oxycodone drug administration

57 mice were administered oxycodone hydrochloride (CH2 Direct, OxyNorm 50 mg/ml diluted in saline), twice daily (7 h separating injections) for 8 days, at increasing doses of 9, 17.8, 23.7 and 33 mg/kg administered on days 1–2, 3–4, 5–6 and 7–8, respectively. On the 9th day, a single 33 mg/kg dose of oxycodone was administered. 78 control mice were administered saline for 9 days using the same dosing schedule. Oxycodone and saline were administered intraperitoneally (IP) at an injection volume 10 ml/kg. Weights were taken at 4 time points throughout the protocol. The protocol was adapted from Enga et al. (2016).

### Org27569 for precipitated withdrawal

On day 9, 2 h after the final oxycodone injection and immediately before withdrawal testing, 80 mice (40 female) were administered 10 mg/kg naloxone hydrochloride (Sigma Aldrich) IP (injection volume 10 ml/kg) to precipitate withdrawal. Mice received either 0, 3, 10 or 30 mg/kg of Org27569 IP 10 min before naloxone and 10 min prior to testing, consistent with previous studies (Ding et al. 2014; Jing et al. 2014). Org27569 was prepared in a 1:1:18 ratio of DMSO, tween 80, and saline and was administered at an injection volume of 10 ml/kg. Immediately following naloxone administration, mice were placed in acrylic arenas and recorded for 30 min with two side-on cameras (90 fps). The 20(w) x 20(l) x 30(h) cm arena had transparent walls, blue floors, and a soft plastic roof to safely prevent mice escaping. Fecal boli were counted and presence of diarrhea were noted by a blinded experimenter at the end of each test. Mice were weighed before and after the test. Jumps were automatically scored from DeepLabCut (version 2.3; Mathis et al. 2018) body tracking using a custom python script. The DeepLabCut model was trained using > 3,000 manually labelled frames from > 100 videos; 95% of these frames were used to train the pose estimation model using ResNet-50 with default parameters. A likelihood cut-off of *p* < .2 was used to condition the X, Y coordinates and jumping events were annotated based on the velocity of tracking coordinates. This method has been rigorously validated in-house and performs at or better than manual human annotation.

### Open field

Org27569’s effects in the open field were tested in 12 male and 8 female mice in a 40 cm³ opaque blue open top box (lighting measured approximately 20 lx). Mice were habituated to the arena for 10 min, approximately 1 h prior to the open field test. On test day, mice were administered 0, 3, 10, or 30 mg/kg of Org27569 IP (10 ml/kg) then placed in the arena for 30 min. An overhead camera recorded their movements. Distance travelled and time spent in thigmotaxis was automatically quantified using DeepLabCut (trained on 840 frames from 50 videos) and a custom python script. Thigmotaxis was defined as proportion of time the mouse (denoted as the average x-y coordinate of the NeckBase, BodyCentre, and TailBase) was within 20 mm from the wall.

### Conditioned place aversion

Org27569’s effects on acquisition of naloxone-precipitated withdrawal-induced conditioned place aversion were assessed in a separate group of 60 male mice. The apparatus included two chambers (Chamber A and Chamber B), each 15.5 cm(l) x 15 cm(w) x 15.5 cm(h), with a small door separating them (8(w) x10cm(h)). Chamber A consisted of a bright pink silicone textured floor with blue walls and chamber B consisted of blue non-textured floors and yellow dotted walls on a blue background. The chambers were separated by opaque black Perspex with a removable door. Lighting measured approximately 20 lx. The protocol consisted of 3 distinct phases: pre-conditioning, conditioning and testing.

#### Preconditioning

Mice freely explored the open apparatus for 30 min on two consecutive days. For each mouse, the preferred chamber on day two was designated the naloxone-withdrawal paired arena for the proceeding conditioning sessions. There was no significant chamber preference (*p* = .293).

#### Conditioning

Conditioning ran from day 9–12. Mice in the saline-saline and saline-naloxone conditions received 8 days of saline followed by twice daily (7-h apart) saline during conditioning. Mice in the oxycodone-naloxone condition received 8 days of oxycodone according to the regimen described above, followed by twice daily 33 mg/kg oxycodone injections during conditioning. On either day 9 or 10 and either day 11 or 12, mice received vehicle or 3 mg/kg Org27569 (the dose that did not have any effects on locomotion in the open field) three hours and fifty minutes after their morning oxycodone or saline injection; 10 min later mice received saline (saline-saline group) or 0.6 mg/kg naloxone and were placed into their preferred chamber for 30 min. On the other two days of the conditioning phase, all mice were administered saline and placed in their non-preferred chamber.

#### Testing for expression of a conditioned place aversion

Mice had one abstinence day on day 13, then freely explored the arena with door removed for 30 min on day 14 (CPA test 1). An additional 6-day abstinence occurred between days 15–20 with a final test on day 21 (CPA test 2). Time spent in each chamber was recorded by overhead camera and analysed using a DeepLabCut model trained using ResNet-50 (default parameters) on ~ 500 manually labelled frames from ~ 30 videos, with animal position within the apparatus extracted using python. The dependent variable was the aversion score, calculated as the difference between post-conditioning and pre-condoning time spent in drug-paired compartment (in seconds).

### Elevated plus maze (EPM)

Org27569’s effect on anxiety-like behaviour was assessed in the EPM on day 22 in mice undergoing protracted withdrawal and in control mice. Mice received 0 or 3 mg/kg Org27569 10 min prior to the EPM test. Mice were placed at the junction of the four arms of the maze, facing a closed arm (7 lx). The EPM was raised 41 cm from the ground, arms were 29.5 cm(l) x 4.5 cm(w), and closed arm walls were 14.5 cm(h). An overhead camera captured the 7-minute session. A DeepLabCut model (trained on 500 frames from 25 videos) and custom python script calculated % time spent in the open arms. Chambers were cleaned with ethanol and F10 between trials.

### 3 Chambered social approach test

The effect of Org27569 on sociability and social novelty was assessed using the 3 chambered social approach test on day 23 in mice undergoing protracted abstinence and control mice. The apparatus was a Perspex rectangular box (60 cm(l) x 40 cm(w) x 22.7 cm(h), approximately 20 lx during testing), divided into three equally sized (20 cm(l) x 40 cm(w)) interconnected chambers with removable doors. The walls and doors were transparent, and the floor was blue. A wire-mesh cage with a black rubber roof is placed at each of the two end chambers. The social stimuli were 16 weight matched male C57BL/6 mice, used up to 3 times per day. Stimulus mice were habituated to the stimulus cages for 15-minutes on test day, 2.5-h before testing. The non-social stimulus was 6 marbles. An overhead camera recorded each session, and time spent in each chamber was analysed by DeepLabCut (trained on 800 frames from 40 videos) and a custom python script.

Mice received either 0 or 3 mg/kg of Org27569 IP and were placed immediately in the centre chamber with the doors closed and allowed to explore for 5-min. The doors were then opened and mice were allowed to explore all chambers for 5 min. In the third stage (social preference test) a stimulus mouse was placed in one of the stimulus cages and non-social stimulus in the other, and the test mouse freely explored for 5 min. In the final stage (social novelty preference test), the non-social stimulus was replaced with another social stimulus and the test mouse continued exploring for 5 more minutes. The dependent variable was the time spent in each outer chamber.

### Novel escape chamber

The escape box assay, adapted from Gamage (2013), featured an empty home cage with a wire ladder leading to an ~ 5 cm escape opening in the clear Perspex top that led to metal bars outside the box (see Figure S1 in supplementary files). Mice received 33.3 mg/kg oxycodone IP injections on days 23 and 24, followed by 0.6 mg/kg Naloxone IP 2 h later on day 24. Mice received either 0 or 3 mg/kg Org27569 IP, 10 min before naloxone and were immediately placed into the arena after naloxone administration. Latency to escape (all 4 paws on the metal bars) was measured, with a 10-minute maximum. A latency of 10 min was recorded for mice that did not escape.

### Statistical analyses

ANOVA with planned contrasts was used to analyse continuous variables with data that were normally distributed and met the assumption of homogeneity of variance. If the data violated assumptions, an Aligned Rank Transform (ART) ANOVA and multifactor contrast procedure was performed (Wobbrock et al. 2011). All discrete variables were analysed using an ART ANOVA and multifactor contrast procedure (Elkin et al. 2021). Diarrhea data were analysed using a z test for column proportions to compare the incidence of diarrhea between each treatment group in SPSS (Version 28). All other statistical analyses were conducted using R studio (version 2021.09.2 Build 382). Significance was set at *p* < .05.

## Results

### Org27569 effects on precipitated withdrawal in male and female mice

**Fig. 3:**
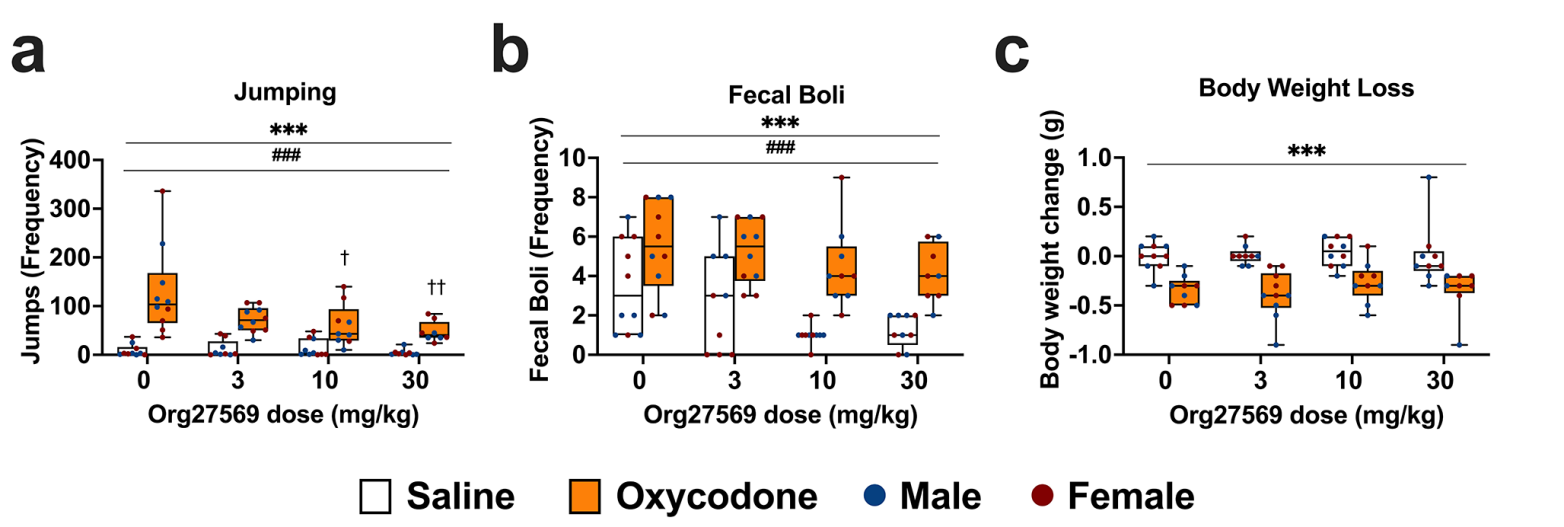
Org27569 reduced naloxone precipitated oxycodone withdrawal induced jumping behaviour in both sexes and reduced gastrointestinal upset in female (but not male) mice, irrespective of whether mice were undergoing withdrawal. **a**: Mice undergoing withdrawal showed increased jumping and Org27569 reduced jumping at the mid and high doses. **b**: Mice undergoing withdrawal showed increased fecal boli and all doses of Org27569 reduced fecal boli in females (but not males) irrespective of whether mice were undergoing withdrawal. **c**: Mice undergoing withdrawal showed increased body weight loss and Org27569 had no effect on body weight loss. ***p < .001, **p < .01, * *p* < .05, main effect of withdrawal. ^###^*p* < .001, ^##^*p* < .01, ^#^*p* < .05, main effect of Org27569 treatment. †††*p* < .001, ††*p* < .01,†*p* < .05, vs. control mice treated with saline. Box extends from 25th to 75th percentile, line represents the median, whiskers extend from min to max, dots represent individual data points. *n* = 8–10

*Jumps* Jump frequency was analysed with ART as it is a discrete variable and data violated assumptions of normality and homogeneity of variance. Mice undergoing PW jumped more frequently than control mice [F(1,59) = 117.01, *p* < .001]. There was a main effect of Org27569 treatment [F(3,59) = 5.80, *p* = .001] and interaction between treatment and withdrawal [F(3,59) = 4.74, *p* = .005]. Org27569 inhibited precipitated withdrawal induced jumps relative to VEH at the mid [10 mg/kg, *p* = .013] and high [30 mg/kg, *p* = .004] doses, but not the low dose [3 mg/kg, *p* = .154]. The effect of Org27569 on jumping was significantly greater in mice undergoing withdrawal than mice not undergoing withdrawal [10 mg/kg, *p* = 005; 30 mg/kg, *p* = .001], with Org27569 having no significant effect on jumping in control mice at any dose [all *p* > .05]. There was no significant difference in withdrawal-induced jumping between the 10 mg/kg and 30 mg/kg dose [*p* = .61]. There was no main effect of sex [F(1,59) = 0.56, *p* = .457] or interaction between sex and withdrawal [*p* = .859] or sex and treatment [*p* = .109]. Jumps data are shown in Fig. 3a.

*Fecal boli* Fecal boli count was analysed with ART as it is a discrete variable and the data violated assumptions of homogeneity of variance. Mice undergoing PW produced more fecal boli than control mice [F(1,59) = 53.51, *p* < .001]. There was a main effect of Org27569 treatment [F(3,59) = 5.72, *p* = .002] and an interaction between treatment and sex [F(3,59) = 4.26, *p* = .009]. Averaged over withdrawal condition, Org27569 treatment reduced fecal boli at all doses tested in females [3 mg/kg, *p* = .016; 10 mg/kg, *p* = .015; 30 mg/kg, *p* = .028], but had no significant effect in males at any dose [all *p* > .05], although the difference in the effect of Org27569 between males and females was only significant at the low dose of Org27569 [*p* = .001]. There was no main effect of sex [F(1,59) = 0.48, *p* = .493] or interaction between withdrawal and sex [*p* = .663] or withdrawal and Org27569 treatment [*p* = .867], suggesting the effects of Org27569 treatment on fecal boli were independent of whether the mice were undergoing withdrawal. Fecal boli data are shown in Fig. 3b.

*Body weight* ART was used to analyse body weight loss as the data violated assumptions of normality and homogeneity of variance. Mice undergoing PW lost more body weight over the test session than control mice [F(1,58) = 97.97, *p* < .001]. There was no main effect of Org27569 treatment [F(3,58) = 0.83, *p* = .484], sex [F(1,58) = 3.98, *p* = .051], and no interactions between withdrawal and sex [*p* = .185], withdrawal and Org27569 treatment [*p* = .878], sex and Org27569 Treatment [*p* = .097]. Body weight data are shown in Fig. 3c.

*Diarrhea* Vehicle treated male, but not female, mice undergoing PW had higher incidence of diarrhea than vehicle treated control mice [Males; *p* < .05, Females; *p* > .05]. Org27569 had no effect on precipitated withdrawal induced diarrhea in either sex [all *p* > .05]. The lowest dose of Org27569 increased the incidence of diarrhea in male control mice [*p* < .05], whereas all other doses had no effect [all *p* > .05]. The percentage of mice within each condition that had diarrhea during the test session is presented in Table 1.

**Table 1:** Percentage of mice with diarrhea in each condition

	Male		Female	
	Vehicle	Oxycodone	Vehicle	Oxycodone
0 mg/kg Org27569	0	80	20	80
3 mg/kg Org27569	100	100	0	100
10 mg/kg Org27569	0	80	20	100
30 mg/kg Org27569	20	100	25	100

Male, but not female, mice undergoing naloxone precipitated oxycodone withdrawal had a higher incidence of diarrhea than untreated mice. *Org27569* had no effect on precipitated withdrawal induced diarrhoea in either sex, however, the lowest dose increased the incidence of diarrhoea in male control mice. *n* = 8-10

### Org27569 *effects on locomotion* and thigmotaxis

**Fig. 4:**
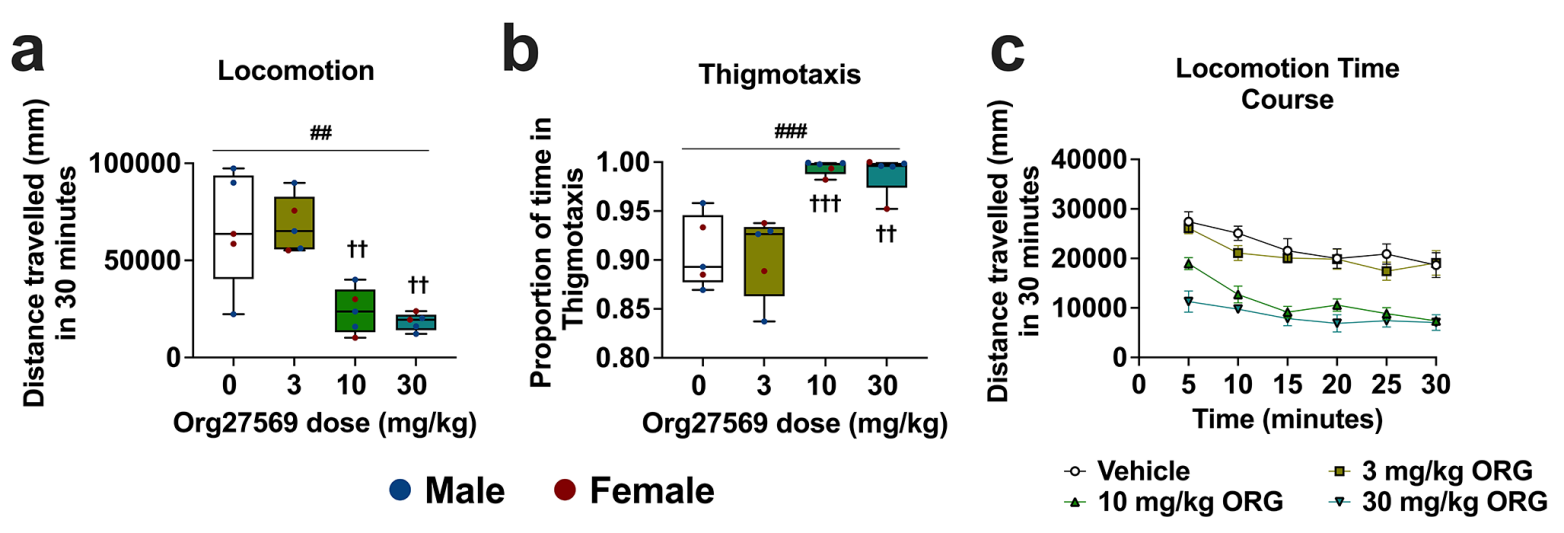
Org27569 reduced distance travelled and increased thigmotaxis in the open field over a 30-minute test session at the mid and highest dose, but not at the lowest dose. **a**: Summary locomotion data **b**: summary thigmotaxis data. **c**: Time course locomotion data, separated into in 5-minute bins. ^##^*p* < .01, main effect of Org27569 treatment. †††*p* < .001, ††*p* < .01, vs. 0 mg/kg Org27569. Box extends from 25th to 75th percentile, line represents the median, whiskers extend from min to max, dots represent individual data points. *n* = 5

Distance travelled and proportion of time spent in thigmotaxis was analysed with ART as data violated the assumption of homogeneity of variance. Averaged over sex, Org27569 treatment had a significant effect on locomotion [F(3, 12) = 6.185, *p* = .009 and thigmotaxis [F(3, 12) = 14.626, *p* < .001], however, averaged over Org27569 treatment condition, there was no effect of sex on locomotion [F(1, 12) = 0.405, *p* = .536] or thigmotaxis [F(1, 12) = 0.197, *p* = .897]. Averaged over sex and compared to vehicle, Org27569 significantly reduced locomotion and increased thigmotaxis at the highest [locomotion; *p* = .008, thigmotaxis; *p* = .002] and mid dose [locomotion; *p* = .013, thigmotaxis; *p* < .001], but not the lowest dose [locomotion; *p* = .978, thigmotaxis; *p* = .882]. There was no difference in the magnitude of effect on locomotion or thigmotaxis between the highest and mid dose [locomotion; *p* = .806, thigmotaxis; *p* = .310]. Locomotion data are shown in Fig. 4.

### Org27569 for the acquisition of conditioned place aversion to precipitated withdrawal and opioid withdrawal induced escape behaviour

*CPA* CPA data were analysed with ANOVA as they were normally distributed and did not violate the assumption of homogeneity of variance. Averaged over Org27569 treatment condition, there was a significant effect of withdrawal condition on CPA scores, one day [F(2,54 = 9.600, *p* < .001] and one week [F(2, 54) = 17.05, *p* < .001] into withdrawal. Averaged over withdrawal condition, there was no effect of 3 mg/kg Org27569 treatment on CPA scores one day [f(1,54) = 1.998, *p* = .163] or one week [f(1,54) = 1.10, *p* = .299] into withdrawal and there was no interaction between withdrawal condition and treatment condition one day [*p* = .776] or one week [*p* = .622] into withdrawal.

**Fig. 5:**
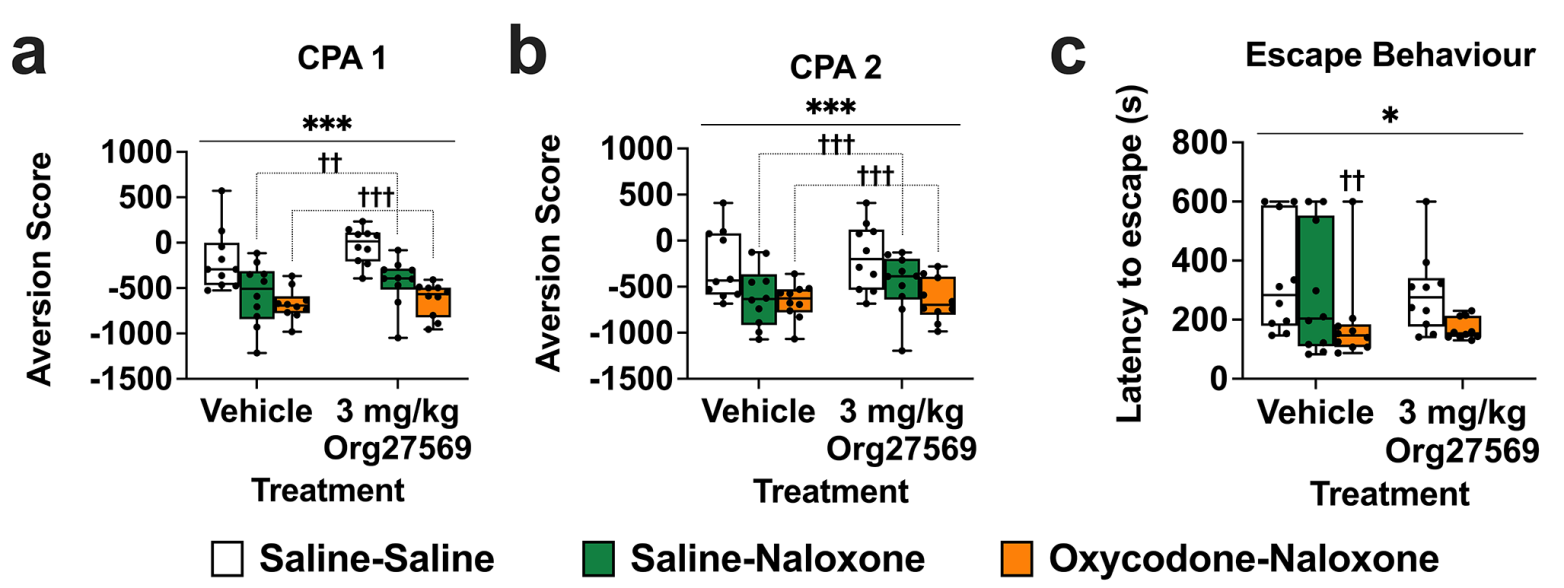
3 mg/kg Org27569 had no effect on naloxone precipitated oxycodone withdrawal induced conditioned place aversion or escape behaviour in mice. Aversion score: difference between post-conditioning and pre-condoning time spent in drug-paired compartment. **a**: One day into abstinence, naloxone elicited a significant aversion in both saline exposed and oxycodone exposed mice. **b**: One week into abstinence, naloxone elicited a significant aversion in both saline exposed and oxycodone exposed mice. **c**: Naloxone reduced the latency to escape in oxycodone but not saline exposed mice. ***p < .001, * *p* < .05, main effect of withdrawal condition. †††*p* < .001, ††*p* < .01, vs. saline-saline. Box extends from 25th to 75th percentile, line represents the median, whiskers extend from min to max, dots represent individual data points. *n* = 10

Averaged over treatment condition, both chronic saline and chronic oxycodone mice administered naloxone elicited a significant CPA one day into withdrawal compared to mice that received saline [saline-naloxone vs. saline-saline; *p* = .002; oxycodone-naloxone vs. saline-saline, *p* < .001]. The effect of naloxone on CPA scores did not differ between chronic saline treated and chronic oxycodone treated mice, *p* = .095. Averaged over treatment condition, chronic saline and chronic oxycodone mice administered naloxone elicited a significant CPA one week into withdrawal compared to mice that received saline [saline-naloxone vs. saline-saline; *p* < .001; oxycodone-naloxone vs. saline-saline, *p* < .001]. The effect of naloxone on CPA scores did not differ between chronic saline treated and chronic oxycodone treated mice, *p* = .928. Data are shown in Fig. 5a and b.

*Escape Behaviour* ART was used to analyse latency to escape as data violated the assumptions of normality and homogeneity of variance. There was a significant effect of withdrawal condition on latency to escape [F(4, 44) = 2.904, *p* = .032]. Vehicle treated mice undergoing precipitated oxycodone withdrawal had a reduced latency to escape compared to vehicle treated control mice [vehicle-oxycodone-naloxone vs. vehicle-saline-saline, *p* = .008]. Naloxone had no significant effect on latency to escape in control mice not dependent on oxycodone [vehicle-saline-naloxone vs. vehicle-saline-saline, *p* = .147]. Org27569 (3 mg/kg) had no effect on latency to escape in control mice not undergoing withdrawal [saline-saline-saline vs. saline-saline-Org27569, *p* = .815], or in mice undergoing precipitated withdrawal [oxycodone-naloxone-saline vs. oxycodone-naloxone-Org27569, *p* = .571]. Escape behaviour data are shown in Fig. 5c.

### Org27569 effects on anxiety like behaviour, sociability, social novelty during protracted withdrawal

**Fig. 6:**
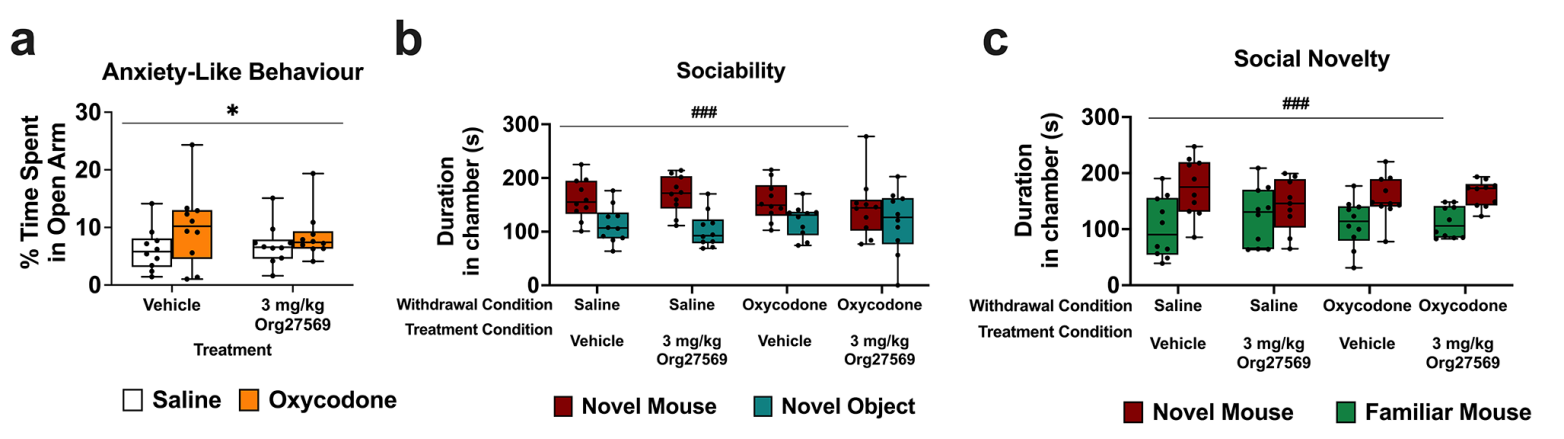
3 mg/kg Org27569 had no effect on anxiety-like behaviour, sociability or social novelty.**a**: mice undergoing withdrawal showed reduced anxiety like behaviour compared to mice not undergoing withdrawal, but Org27569 had no effect on anxiety like behaviour. **b**: Neither withdrawal or Org27569 treatment had any effect on sociability. **c**: Neither withdrawal or Org27569 treatment had any effect on sociability. *p < .05, main effect of withdrawal condition, ^###^*p* < .001, main effect of chamber side. Box extends from 25th to 75th percentile, line represents the median, whiskers extend from min to max, dots represent individual data points. *n* = 10

*EPM test* EPM data violated the assumptions of normality, therefore, ART was used. Mice undergoing protracted withdrawal spent a significantly greater percentage of time in the open arms than mice not undergoing withdrawal [(F1, 36) = 5.06, *p* = .031]. There was no effect of 3 mg/kg Org27569 treatment on the percentage of time spent in the open arms [F(1,36] = 0.43, *p* = .516] and no interaction between withdrawal condition and treatment [*p* = .230]. EPM data are shown in Fig. 6a.

*3 chambered social approach test* Data were normally distributed and did not violate the assumption of homogeneity of variance, as such ANOVA was used. Mice spent more time in the chamber with the novel mouse than the novel object [F(1, 72) = 22.048, *p* < .001]. There was no effect of withdrawal condition time spent investigating the outer chambers [F(1, 72) = 0.0249, *p* = .876], no effect of 3 mg/kg Org27569 treatment [F(1, 72) = 0.1499, *p* = .670], and no interaction between withdrawal and chamber [*p* = .164] or between treatment and chamber [*p* = .822].

Averaged over withdrawal and treatment condition, mice spent more time in the chamber with the novel mouse than the familiar mouse [F(1, 71) = 23.216, *p* = < 0.001], showing normal social novelty preference. Averaged over Org27569 treatment, there was no effect of withdrawal condition on time spent in each chamber [F(1, 71) = 0.0001, *p* = .990] and averaged over withdrawal condition, there was no effect of 3 mg/kg Org27569 treatment on time spent in each chamber [F(1, 71) = 0.003, *p* = .959], and no interaction between withdrawal and chamber [*p* = .909] or between treatment and chamber [*p* = .236].

Sociability and social novelty data are shown in Fig. 6b and c, respectively.

## Discussion

This is the first study to examine the effects of CB1R NAM Org27569 on acute oxycodone withdrawal and protracted abstinence from oxycodone in mice. Org27569 reduced opioid withdrawal-induced jumping at the 10 and 30 mg/kg doses, but not the 3 mg/kg dose. Additionally, at all doses tested, Org27569 had a modest inhibitory effect on gastrointestinal motility in female mice, reducing fecal boli output irrespective of withdrawal status, but had no impact on the incidence of diarrhea or withdrawal-induced body weight loss. Notably, sex difference should be interpreted with caution due to the low sample size for each sex. The 10 and 30 mg/kg doses of Org27569 reduced locomotion and increased the proportion of time spent in thigmotaxis, indicating that these doses are sedating or anxiogenic, thus the 3 mg/kg dose was used for subsequent experiments to avoid confounding locomotor effects. At 3 mg/kg, Org27569 had no effect on the acquisition of precipitated withdrawal-induced CPA measured at 24 h or 7 days post-conditioning. In the escape box test, mice undergoing precipitated withdrawal had a shorter latency to escape, supporting this test as a model of goal directed escape behaviour elicited by opioid withdrawal. 3 mg/kg Org27569 had no effect on latency to escape. During protracted abstinence from opioids, decreased anxiety-like behaviour was observed compared to control mice, but sociability and social novelty did not differ, suggesting mice did not experience a pronounced protracted withdrawal syndrome in the present study. 3 mg/kg Org27569 had no effect on behaviour in the EPM or social behaviour in the tests conducted during protracted abstinence, however, the lack of a robust withdrawal phenotype clouds the interpretation of these null effects. Taken together, although Org27569 reduced jumping behaviour associated with opioid withdrawal at doses of 10 and 30 mg/kg, these effects were confounded by reduced locomotion, with the lower dose having no opioid withdrawal-specific effects. These data therefore question the potential utility of Org27569 for the treatment of acute opioid withdrawal symptoms and further studies are needed in a model that produces a more pronounced protracted withdrawal syndrome.

The reduction in opioid withdrawal-induced jumping in response to negative allosteric modulation of the CB1R with Org27569 is consistent with previous research by (Trang et al. 2006) who showed that the CB1R inverse agonist rimonabant reduced naloxone-precipitated morphine withdrawal-induced jumping behaviour. However, the efficacious doses of Org27569 in the present study reduced locomotion, suggesting the reduced jumping may be driven by non-specific locomotor effects rather than specific effects on the withdrawal-induced negative affective state driving the jumping (Azizi et al. 2012). Locomotor effects of Org27569 have not been reported previously, although the Org27569 analogue, LDK1258 was shown to reduce locomotion at 30 mg/kg via a non-CB1R-mediated mechanism (Mustafa et al. 2020). Further, significant thigmotaxis was observed at mid and high doses in the open field, therefore, the reduction in locomotion could be due to anxiogenic effects of Org27569, rather than a sedative effect. However, it is difficult to interpret thigmotaxis in the context of reduced locomotion (La-Vu et al. 2020). Given we did not observe any anxiogenic like effects of Org27569 on the EPM, it is more likely that Org27569 is having a sedating than anxiogenic effect, however future studies might examine other assays of locomotion, such as the rotor rod, to further explore this interpretation.

In addition to its impact on withdrawal-induced jumping behaviour, Org27569 exhibited a modest inhibitory effect on gastrointestinal motility at all three doses tested, reducing fecal boli regardless of withdrawal condition. These findings suggest that the effect is not specific to opioid-related gastrointestinal symptoms and are consistent with the extensive expression of CB1Rs in the enteric nervous system (Pertwee 2001). Interestingly, these results parallel our previous work which demonstrated a similar, non-specific effect of CBD on gastrointestinal symptoms in the same oxycodone withdrawal model, thus providing a potential mechanism for CBD’s effects, given one of CBDs mechanisms is CB1R NAM (Laprairie et al. 2015). Notably, the effect of CBD on gastrointestinal symptoms was more pronounced than that of Org27569 in the current study.

Org27569 (3 mg/kg) had no effect on the acquisition of CPA to oxycodone withdrawal, naloxone precipitated withdrawal-induced escape behaviour, behaviour in the EPM or social behaviour during protracted abstinence. This is inconsistent with previous studies supporting the effectiveness of reducing activity at the CB1R to alleviate opioid withdrawal symptoms. CB1R knock-out (Ledent et al. 1999; Lichtman et al. 2001; Maccarrone et al. 2002) and treatment with CB1R inverse agonist rimonabant both reduced morphine withdrawal severity in rodent models (Rubino et al. 2000; Mas-Nieto et al. 2001; Trang et al. 2006; Bergman et al. 2008), while CB1R antagonists blocked the acquisition of conditioned place aversion to morphine withdrawal in rats (Wills et al. 2014, 2017; Wills and Parker 2016). Additionally, cannabidiol (CBD), a CB1R NAM, alleviated opioid withdrawal symptoms in mice, including gastrointestinal upset and anxiety-like behaviour (Bhargava 1976; Navarrete et al. 2022; Scicluna et al. 2022); although CBD acts at multiple targets (Martin et al. 2021), so these effects may be CB1R independent. It is therefore possible that CB1 NAM is insufficient to recapitulate the effects on opioid withdrawal symptoms observed with CB1R knockout, inverse agonists, and antagonists.

An alternative explanation is that the Org27569 compound may lack suitable potency and selectively to have clear and specific effects on opioid withdrawal symptoms. It is possible that the 3 mg/kg dose was too low, however, previous studies have shown in vivo efficacy at similar doses in rats. For example, a dose of 3.2 mg/kg has been shown to reduce the hypothermic actions of both CP55940 and anandamide (both CB1 receptor agonists) and attenuate cue and drug-induced reinstatement of cocaine and meth seeking behaviours (Ding et al. 2014; Jing et al. 2014). However, Ding et al. (2014) found that a dose of 3.2 mg/kg did not decrease the intake of palatable food, while higher doses (5.6 and 10 mg/kg) did. It is also possible that 3 mg/kg is not sufficient to inhibit these behaviours in mice but could be effective in rats. The higher doses of Org27569 (10 and 30 mg/kg) were effective at reducing opioid withdrawal-induced jumps, consistent with the literature; however, these were accompanied by confounding sedation, possibly mediated by off-target effects, suggesting that there is no strong rationale to use higher doses when attempting to selectively target the CB1 receptor. Off target effects have been observed previously; Org27569 reduced food intake in mice through non-CB1 receptor mechanisms (Gamage et al. 2014). Finally, it is also possible that Org27569 has questionable utility as a CB1 NAM in vivo. Its effects on other CB1R-mediated actions are not well-established, and its utility as CB1 NAM in rodents has been questioned previously (Ding et al. 2014; Gamage et al. 2014). While Org27569 has been shown to reduce food intake and drug-seeking behaviour in rodents, it lacked efficacy in altering antinociceptive, cataleptic, and hypothermic actions of the orthostatic agonists anandamide, CP55,940, and Δ9-tetrahydrocannabinol (Ding et al. 2014; Gamage et al. 2014; Jing et al. 2014). Future studies with more potent CB1R NAMs with a more favourable off-target profile would help to address whether Org27569 specifically, or CB1R NAM more broadly, is the issue.

Beyond an evaluation of Org27569 during opioid abstinence, the current study offers insights into the negative affective symptoms associated with acute and protracted abstinence from oxycodone in mice. Naloxone-precipitated withdrawal resulted in a pronounced CPA that was present from 24 h to at least one week into abstinence. However, naloxone also elicited a CPA in opioid naïve mice, and the difference in the effects of naloxone in opioid naïve and opioid dependent mice only approached significance. This suggests naloxone was a major driver of the aversion, raising questions about the robustness of the CPA effect in this experiment. It’s unclear why a low dose of 0.6 mg/kg naloxone did not produce a stronger aversion in oxycodone dependent mice compared to saline exposed mice. Previous studies have shown a stronger aversion in morphine dependent mice compared to naïve mice at doses of morphine that were lower, equivalent, and higher than the doses of oxycodone used in this study (Blokhina et al. 2000; Shoblock and Maidment 2006; Garcia-Carmona et al. 2012; Gomez-Milanes et al. 2012; Gamage et al. 2015; Wang et al. 2016; Navarro-Zaragoza et al. 2021). We calculated the oxycodone equivalent dose using the conversion factor described in Von Korff et al. (2008). However, in the one study that examined CPA to oxycodone withdrawal, they did not include an opioid naïve naloxone group (Olmstead and Burns 2005).

It may simply be that CPA models are especially sensitive to the effects of naloxone, as in the present study the same 0.6 mg/kg dose of naloxone used in the CPA model elicited escape behaviour in opioid dependent mice, but not in opioid naïve mice, suggesting that dose of naloxone does induce oxycodone withdrawal specific effects. This was established using a novel escape box assay adapted from Gamage (2013). Escape in this assay and jumping in the standard opioid withdrawal test are both measures of escape behaviour, a negative affective symptom of opioid withdrawal. Withdrawal-induced jumps depend on the height of the chamber (Azizi et al. 2012), suggesting it is a goal directed escape behaviour. The escape box assay used in the present study more precisely captures the goal directed nature of escaping from an aversive environment, thus it may be a superior model of negative affect during opioid withdrawal than measuring withdrawal-induced jumping.

In this study, mice undergoing protracted abstinence from oxycodone showed less anxiety-like behaviour on the EPM. This could be due to “adaptive coping” following chronic stress, previously observed in C57BL/6J mice (Mozhui et al. 2010; Bravo et al. 2020). Our finding is consistent with a previous study that reported reduced anxiety-like behaviour on the EPM in C57BL/6J mice 6 weeks into morphine abstinence (Bravo et al. 2020). In contrast, another study found no influence of 28 days of oxycodone abstinence on anxiety-like behaviour in C57BL/6 mice in the marble burying test (Sanchez et al. 2016). An alternative explanation is that the increased time spent in the open arm signifies escape behaviour, serving as an indicator of negative affect. Future research should investigate the use of escape behaviour, including use of the escape box, as an indicator of negative affect during protracted withdrawal. For a more nuanced understanding of anxiety-like behaviour during withdrawal, future studies should consider alternative testing paradigms that are not dependent on exploratory behaviour, such as the marble burying test (Becker et al. 2017).

Normal sociability and social novelty preference were observed, regardless of withdrawal or Org27569 treatment. While studies have shown social deficits during protracted morphine withdrawal (for review see Welsch et al. 2020), none have explored social behaviour during protracted oxycodone abstinence. Aside from the increased time spent exploring the open arm in the EPM, which warrants further exploration, the present findings suggest modifications to the protocol may be required to produce a more pronounced protracted withdrawal syndrome. This could include a longer duration of oxycodone exposure or putting mice through repeated cycles of abstinence and withdrawal, which has been shown to produce protracted withdrawal-like behaviours with other substances of abuse (Becker 1998; Bowen et al. 2022). It is also possible that we missed the window for protracted withdrawal induced social deficits or that sociability and social novelty are unreliable measure for assessing protracted oxycodone withdrawal in mice and other measures should be explored.

This is the first study to examine the effects of Org27569 during acute and protracted abstinence from opioids. Although Org27569 reduced opioid withdrawal-induced jumping at doses of 10 and 30 mg/kg, these effects were confounded by reduced locomotion. At the lower dose of 3 mg/kg, which was not confounded by locomotor effects, Org27569 had no effect on naloxone-precipitated withdrawal induced jumping, acquisition of oxycodone withdrawal-induced CPA, naloxone precipitated withdrawal-induced escape behaviour in a novel assay, opioid withdrawal-induced alterations in anxiety like behaviour, sociability, or social novelty preference. These data question the utility of Org27569 for treating opioid withdrawal symptoms, and studies with more potent and selective CB1R NAMs are needed to determine with NAM of the CB1R has potential utility for treating opioid withdrawal. Further studies are needed in models that produce a more pronounced protracted opioid withdrawal syndrome.

### Electronic supplementary material

Below is the link to the electronic supplementary material.

Supplementary Material 1
