# Correction to: Effects of CB1 receptor negative allosteric modulator Org27569 on oxycodone withdrawal symptoms in mice

**DOI:** 10.1007/s00213-024-06605-w

**Published:** 2024-05-06

**Authors:** Rhianne L. Scicluna, Nicholas A. Everett, Connie J. Badolato, Bianca B. Wilson, Michael T. Bowen

**Affiliations:** 1 Brain and Mind Centre, The University of Sydney, 94 Mallet Street, Camperdown, Sydney, NSW 2050 Australia; 2 Faculty of Science, School of Psychology, The University of Sydney, Sydney, NSW 2006 Australia

**Correction to: Psychopharmacology**

10.1007/s00213-024-06591-z

*Diarrhea* Vehicle treated male, but not female, mice undergoing PW had higher incidence of diarrhea than vehicle treated control mice [Males; *p* < .05, Females; *p* > .05]. Org27569 had no effect on precipitated withdrawal induced diarrhea in either sex [all *p* < .05]. The lowest dose of Org27569 increased the incidence of diarrhea in male control mice [*p* > .05], whereas all other doses had no effect [all *p* > .05].

The “<” and “>” symbols in the text should be “>” and “<” respectively.

It should read:

*Diarrhea* Vehicle treated male, but not female, mice undergoing PW had higher incidence of diarrhea than vehicle treated control mice [Males; *p* < .05, Females; *p* > .05]. Org27569 had no effect on precipitated withdrawal induced diarrhea in either sex [all *p* > .05]. The lowest dose of Org27569 increased the incidence of diarrhea in male control mice [*p* < .05], whereas all other doses had no effect [all *p* > .05].

The original article has been corrected.

