# Supplementary figures and images for: Effects of CB1 receptor negative allosteric modulator Org27569 on oxycodone withdrawal symptoms in mice
Source: Psychopharmacology (Berl). 2024 Apr 27;241(8):1705–17. doi: 10.1007/s00213-024-06591-z (PMC11269377; doi:10.1007/s00213-024-06591-z)

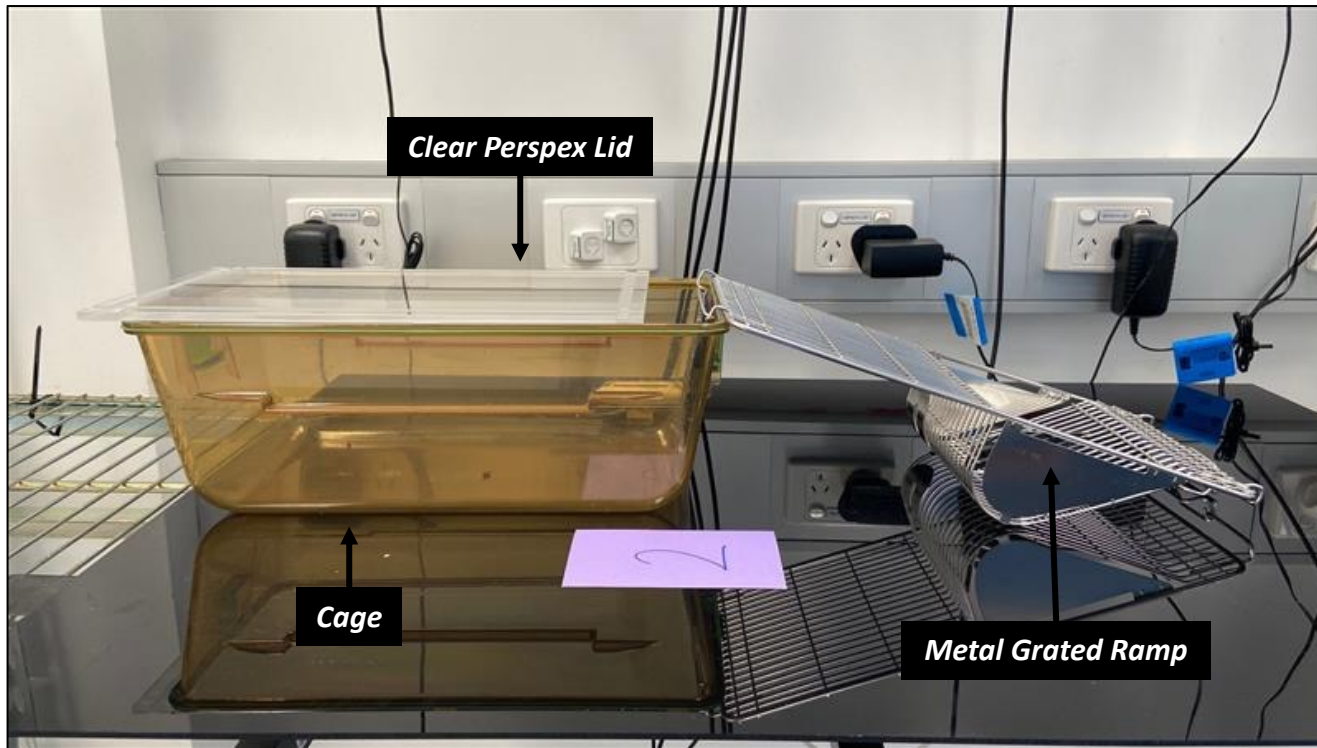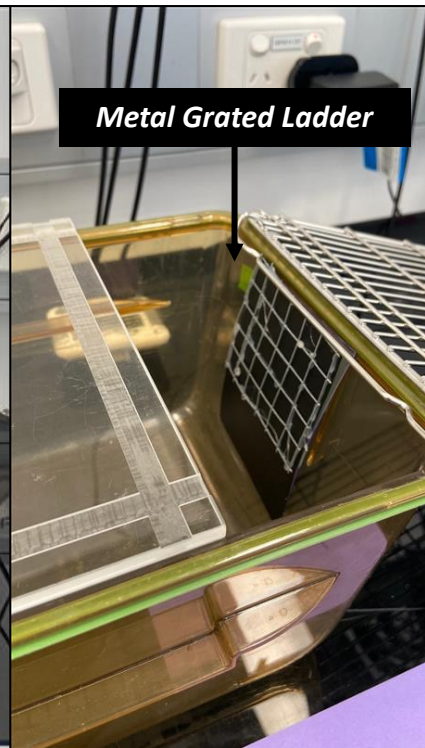

Supplement: Supplementary file 1 — Supplementary Material 1 [file 213_2024_6591_MOESM1_ESM.pdf]
